# Up cycling prickly pear peel waste for sustainable wool dyeing using microwave irradiation

**DOI:** 10.1038/s41598-026-55128-1

**Published:** 2026-06-08

**Authors:** Lamiaa K. El Gabry, Thanaa F. Abdelhafez, Osama A. Hakeim

**Affiliations:** 1 Protinic and Man-made fibres Department, Textile Research and Technology Institute, National Research Centre, Cairo 12622 Dokki, Egypt; 2 Dyeing, Printing, and Textile Auxiliaries Department, Textile Research and Technology Institute, National Research Centre, Dokki, 12622 Cairo Egypt

**Keywords:** Prickly pear peel waste, Natural dye, Betalain pigments, Wool fabric, Microwave-assisted dyeing, UPF protection, Antibacterial activity, Sustainable textile processing, Chemistry, Environmental sciences, Materials science

## Abstract

This study explores the valorization of prickly pear (Opuntia ficus-indica) peel waste as a sustainable natural dye for wool fabrics. Dyeing was conducted using both conventional mordant-assisted (4% tannic acid) and mordant-free microwave-assisted methods. Key parameters including dyeing time and pH were optimized to enhance color strength. Microwave-assisted dyeing substantially reduced processing time and energy consumption compared to the conventional method. The dyed fabrics were evaluated for mechanical properties, color strength, fastness properties, ultraviolet protection factor (UPF), and antibacterial activity. Microwave-dyed samples exhibited higher color strength, improved fastness ratings, higher UPF values, and better antibacterial activity than conventionally dyed samples. In addition, the microwave process produced effluent with lower BOD, COD, and TDS levels. These findings indicate that prickly pear peel extract combined with microwave technology offers a promising eco-friendly approach for producing colored wool fabrics with added functional properties while reducing environmental impact.

## Introduction

The textile industry, a significant global economic contributor, faces increasing pressure to adopt sustainable practices due to the environmental impact of conventional dyeing processes. Traditional synthetic dyes often involve hazardous chemicals and generate substantial wastewater, posing considerable ecological challenges^1^. This has spurred a growing interest in natural dyes as a viable and eco-friendly alternative, aligning with the principles of green chemistry and circular economy^2^.

**Fig. 1:**
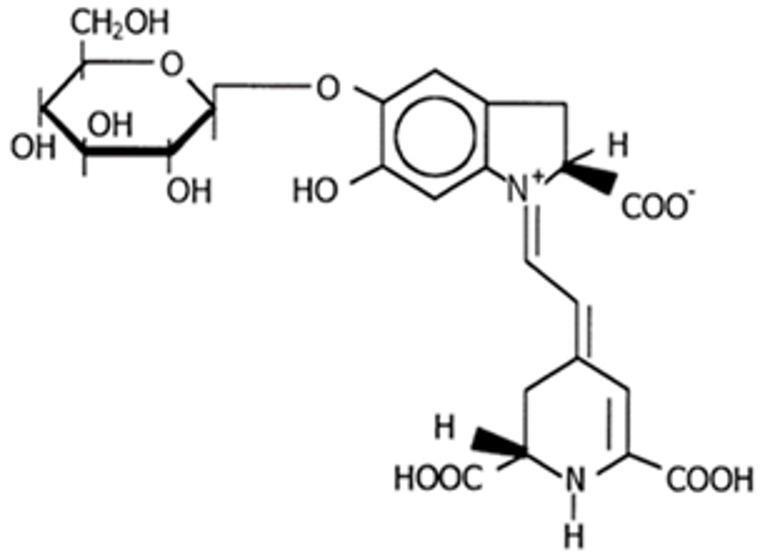
Molecular structure of betanin.

Natural dyes, derived from various biological sources such as plants, insects, and minerals, offer biodegradability, non-toxicity, and often impart additional functional properties to textiles, including antimicrobial activity and UV protection^3^. Prickly Pear Waste as a Sustainable Dye Source Among the diverse natural sources, agricultural waste streams present a particularly promising avenue for sustainable dye production. The valorization of such waste not only reduces environmental burden but also adds economic value to agricultural by-products. Prickly pear (Opuntia ficus-indica (L.) Miller), a cactus species widely cultivated in arid and semi-arid regions globally, is renowned for its edible fruits. However, the peels of these fruits, often discarded as waste, are rich in betalain pigments (Fig. 1), which are natural, water soluble colorants responsible for the vibrant red-purple and yellow hues1. These betalains, along with other polyphenolic compounds like flavonoids, also contribute antioxidant and anti-inflammatory properties, making prickly pear peel waste a valuable resource for natural dye extraction1. Previous research has extensively explored the potential of prickly pear peel waste in various industrial and environmental applications. For instance, Scarano et al. (2020) explored the extraction of natural dyes from prickly pear peels, identifying a rich chemical profile of bioactive compounds suitable for dyeing linen, cotton, and wool fabrics, achieving vibrant hues with good color fastness1. More recently, Barani and Rahmani (2023) utilized aqueous extracts from Opuntia ficus-indica fruit peels for wool yarn dyeing, employing ascorbic acid as a bio-mordant to enhance color strength and uniformity under varied pH and temperature conditions2. Similarly, Riaz et al. (2023) investigated prickly pear peel juice as a pollution-free betalain source for wool yarn, optimizing extraction and dyeing parameters to yield eco-friendly, antimicrobial-colored textiles^4^. In a 2025 study, Ali et al. synthesized metal-complex dyes from red prickly pear peels by adding ions like Cu, Co, and Ni, applying them to wool via microwave and conventional methods, resulting in enhanced color performance and functional properties^5^. Additionally, a 2024 study by Mureddu et al. reported the use of polysaccharides extracted from prickly pear peels as natural additives for textile dyeing, improving dye fixation and sustainability in cotton and wool applications^6^. Beyond coloration, prickly pear peel waste has been repurposed in other applications, further emphasizing its versatility. For example, it serves as an effective biosorbent for removing textile dyes and pollutants from wastewater, as shown in studies where powdered peels exhibited high adsorption capacity for phenols and nitrophenols^7^^[,8^. In food science, peel by-products have been used as natural antioxidants in margarines, extending shelf life while reducing synthetic additive reliance^9^^[,10^. Recent innovations include upcycling peels for nanomaterial synthesis, such as ZnO nanoparticles for foodborne pathogen control^11^, and as fillers in polylactic acid (PLA)-based biocomposites for enhanced mechanical properties and waste reduction^12^. These multifaceted applications underscore the role of prickly pear peel waste in promoting circular economy principles across industries.

Wool, a natural protein fiber, has been a staple in textile production for centuries due to its excellent insulation, comfort, and durability. Its unique chemical structure, characterized by polypeptide chains and various functional groups, makes it highly receptive to a wide range of dyes. However, conventional wool dyeing often relies on synthetic dyes and mordants, some of which can be environmentally detrimental^2^^[,13^.

The study involved the application of a new dye, made from natural and synthetic compounds, to impart ultraviolet protection factor (UPF) and antimicrobial properties to wool fabrics. The research included dyeing wool fibers with a dye derived from roadmen, resulting in fibers with enhanced UV resistance and antibacterial activity^14^.

The application of natural dyes to wool fabrics offers an environmentally benign alternative, contributing to the development of sustainable fashion. While natural dyes provide aesthetic appeal and functional benefits, challenges such as lower dye exhaustion, fixation efficiency, and light fastness compared to synthetic dyes have historically limited their widespread industrial adoption. Researchers are actively exploring innovative methods to overcome these limitations, focusing on enhancing dye uptake and colorfastness without compromising environmental integrity^15^^[,2^.

In recent years, advanced dyeing techniques have emerged to improve the efficiency and sustainability of textile coloration^15,17^. Microwave irradiation, a non-conventional heating method, has gained significant attention in the textile industry due to its numerous advantages over traditional heating methods. Microwave energy promotes rapid and uniform heating by directly interacting with polar molecules in the dye bath, leading to accelerated dye diffusion and improved dye uptake^3^. This results in reduced dyeing times, lower energy consumption, and decreased water usage, thereby contributing to a moreneco-friendly dyeing process. Studies have demonstrated that microwave-assisted dyeing can achieve comparable or even superior color strength and fastness properties compared to conventional methods, often without the need for harsh chemicals or excessive mordants^4^^[,17^.

The direct integration of microwave-assisted dyeing with this particular bio-waste-derived dye on wool to achieve a comprehensive, multipurpose finish is still unexplored, despite the fact that prior studies have successfully used prickly pear peel extract for wool dyeing and others have applied microwave technology to textile processes. Thus, the synergistic application of microwave irradiation to valorize prickly pear peel waste for wool dyeing is the main originality of our work. Through mordant-free processing and cleaner effluents, this study uniquely shows that this integrated approach not only significantly improves process efficiency and color strength but also concurrently imparts superior functional properties, particularly “excellent” graded UV protection and significant antimicrobial activity, while achieving a measurable reduction in environmental impact.

The study aims to use prickly pear peel waste (Opuntia ficus-indica) as a sustainable natural dye for wool fabrics, addressing environmental waste from agricultural byproducts. Objectives include extracting betalain pigments for dyeing using conventional and microwave-assisted methods to reduce resource use and optimize dyeing parameters for maximum color strength. The research evaluates color uptake and mechanical properties, tests color fastness and antibacterial activity, and demonstrates environmental benefits by comparing dye bath effluents from both methods. This process promotes eco-friendly textile practices, enhancing wool garments with UV protection and antimicrobial properties while minimizing ecological impact.

## Results and discussions

### Statistical analysis

All quantitative experiments were performed in triplicate (*n* = 3). Results are expressed as mean ± standard deviation (SD). Statistical significance between conventional and microwave-assisted methods was determined using Student’s independent t-test (for two-group comparisons) or one-way ANOVA followed by Tukey’s post-hoc test (for multiple comparisons) at a significance level of *p* < 0.05 using SPSS software (version 26).

### The mechanical properties

The mechanical parameters of the wool materials, such as thickness, weight, and shrinkage, were measured before and after dyeing using both microwave-assisted and conventional procedures (Table 1). The colored samples showed an overall increase in fabric thickness and weight when compared to the undyed wool. The microwave-dyed fabric had a thickness of 1.09 mm and a weight of 261 g/m², but the traditionally colored fabric had higher values of 1.201 mm and 262 g/m². These improvements can be linked to the deposition of dye molecules and probable fiber swelling during the dyeing process, which can fill interstitial spaces and add bulk to the fabric structure. Similar findings have been observed in research on natural dyeing of wool, where dye particle adsorption adds to increased fabric density and thickness by forming a thicker surface layer^25^^[,26^. This impact is more evident in traditional dyeing, most likely due to extended exposure to heat and agitation, which allows for greater penetration and concentration of the prickly pear peel extract components. In terms of shrinkage, undyed wool exhibited no significant change, although microwave-dyed and traditionally dyed samples shrank by 5% and 7.5%, respectively. The increased shrinkage in the conventional procedure may be due to prolonged temperature and mechanical stress in the dye bath, which can promote fiber contraction and felting in wool^27^. In contrast, microwave irradiation provides a shorter processing time and more uniform heating, reducing thermal degradation and retaining fiber integrity to a greater extent^22^. This is consistent with previous research showing that wet processing conditions, such as those used in traditional dyeing, aggravate wool hydrolysis and dimensional changes, especially at high temperatures^27^. Overall, these findings indicate that microwave-assisted dyeing not only improves efficiency but also reduces negative impacts on mechanical qualities when compared to traditional methods, indicating its promise as a sustainable alternative for wool processing.

**Table 1:** Mechanical characteristics of wool fabrics dyed by conventional and microwave techniques with prickly pear peel extract (*n* = 3, mean ± SD).

Samples	Fabric thickness( mm)	Fabric weight (g/m²)	Fabric shrinkage %
Undyed wool	0.95 ± 0.02	260 ± 1.5	–
Dyed wool with microwave	1.09 ± 0.03*	261 ± 2.1	5.0 ± 0.4*
Dyed wool with conventional	1.201 ± 0.04*	262 ± 1.8	7.5 ± 0.6*

(*p* < 0.05 vs. undyed control, t-test)

### Spectroscopic analysis of prickly pear fruit waste extract

**Fig. 2:**
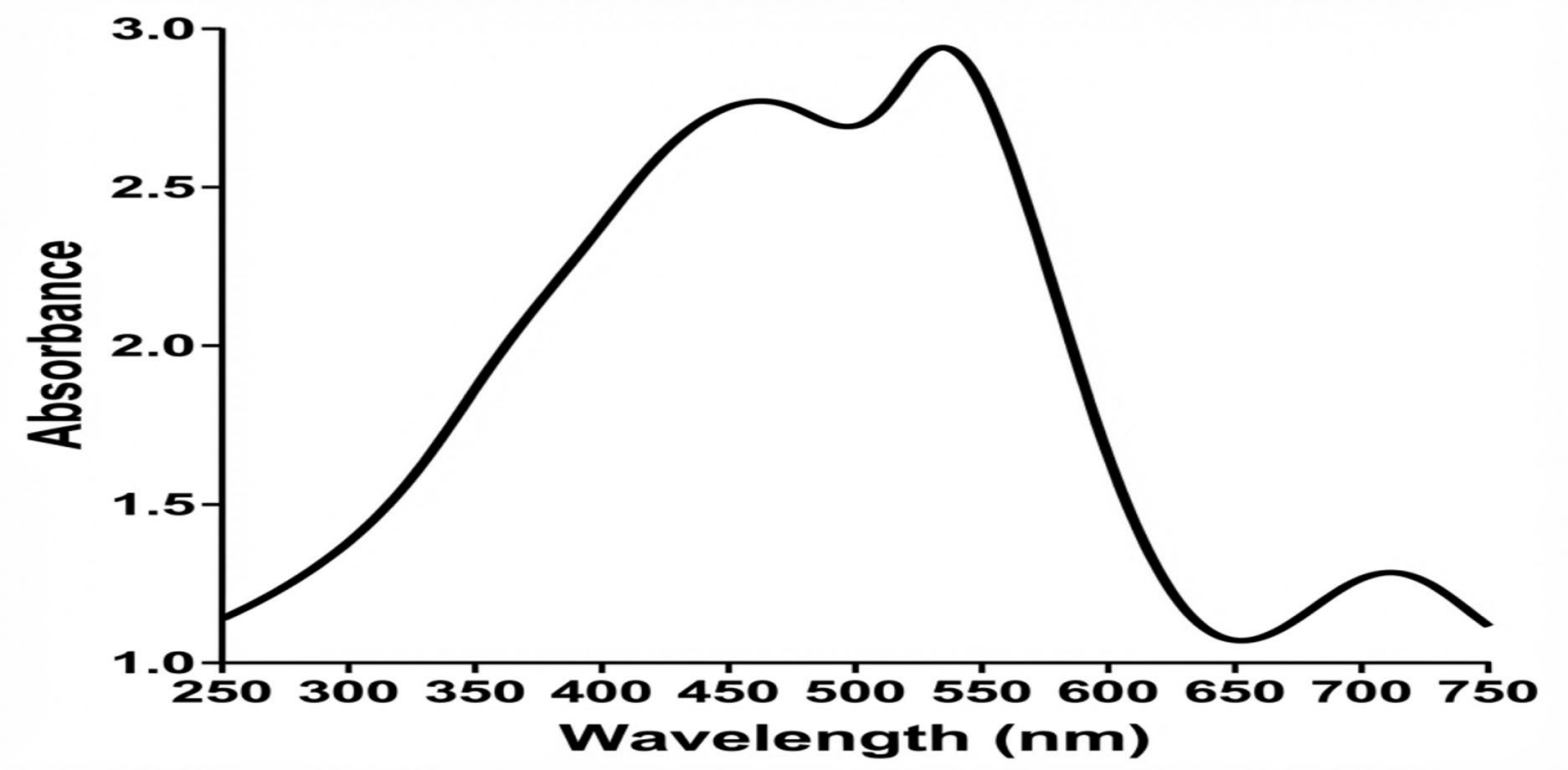
Visible absorption spectrum of the prickly pear peel extract (1:10 dilution) showing λ_max at 480 nm (betaxanthins) and a shoulder at 535–540 nm (minor betacyanins).

The UV-Vis absorption spectrum of the extract from yellow prickly pear fruits (Opuntiaficus-indica), as illustrated in Fig. 2, provides a definitive fingerprint of its constituent pigments. The spectrum is characterized by two prominent absorption maxima in the visible region, which are diagnostic of the two main classes of betalains: betacyanins and betaxanthins^28^. The primary absorption peak observed at approximately 535–540 nm is the characteristic signature of betacyanins, specifically betanin and its isoforms29. These pigments contain a cyclo-DOPA residue conjugated with betalamic acid, which shifts their absorption into the green-yellow part of the visible spectrum, resulting in a red-violet appearance30. The high intensity of this peak in the yellow prickly pear extract confirms that even yellow-orange varieties contain significant amounts of red violet betacyanins, which contribute to the overall depth of shade during the dyeing process. The secondary peak (or shoulder) observed in the 470–480 nm range corresponds to betaxanthins, such as indicaxanthin31. These pigments are formed by the condensation of betalamic acid with amino acids or amines. Their maximum absorption at 480 nm is responsible for the characteristic yellow-orange hue of the fruit31. The presence of both peaks indicates a complex pigment matrix that offers a broader spectral coverage, which is beneficial for achieving varied color shades on wool fabrics18.

Beyond the visible region, while the spectrum in Fig. 1 does not exhibit discrete localmaxima (peaks) within the UV-A (315–400 nm) and UV-B (280–315 nm) ranges, it shows a high and continuously increasing level of absorbance starting from 250 nm. This strong and broad-band UV absorption is a critical factor in the high Ultraviolet Protection Factor (UPF) values observed in the dyed wool fabrics (as discussed in Table 2)32, 33. The phenolic and aromatic structures, including the cyclo-DOPA residue, within the betalain molecules act as efficient UV filters, dissipating the energy of incident UV radiation and protecting both the fiber and the wearer’s skin from photo-damage34. The well-defined nature of the peaks in Fig. 2 suggests that the extraction process (whether conventional or microwave-assisted) successfully preserved the integrity of the betalain chromophores35. Microwave-assisted extraction, in particular, is known to enhance the yield of these pigments by promoting rapid cell wall rupture without the prolonged thermal exposure that can lead to the degradation of heat-sensitive betalains36. The stability of these spectral features across the pH range used in the dyeing process further underscores the technical robustness of prickly pear extract as a natural colorant36. The dual-peak profile of the prickly pear extract reflects a synergistic pigment system where betacyanins and betaxanthins coexist, providing both a rich color palette and inherent functional properties such as UV shielding37.

**Table 2:** Fastness properties of wool fabrics dyed with prickly pear peel extract using conventional and microwave methods

Fabric sample	pH	Washing			Perspiration				Rubbing		Light
		Alt	St_W_	St_S_	Acid		Basic		Dry	Wet	
					St_W_	St_S_	St_W_	St_S_			
Dyed Microwave	2	4-5	4-5	4-5	4	4	4	4	4-5	4	6-7
	5	4-5	4-5	4-5	4-5	4-5	4-5	4-5	4-5	4	6-7
	6	4-5	4-5	4-5	4	4	4	4	4-5	4-5	6
	8	4-5	4-5	4-5	4	4	4-5	4-5	4-5	4-5	7
	12	4	4-5	4	4	4	4	4-5	4-5	4-5	6-7
Dyed Conventional	2	4-5	4-5	4-5	4	4	4-5	4	4	4	6-7
	4	4-5	4-5	4-5	4-5	4-5	4-5	4-5	4-5	4-5	6-7
	6	4-5	4-5	4	4-5	4-5	4	4	4	4-5	6-7
	8	4-5	4	4	4-5	4-5	4-5	4-5	4	4-5	6-7
	12	4-5	4	4-5	4-5	4-5	4-5	4-5	4-5	4-5	7

### FTIR spectroscopic analysis

**Fig. 3:**
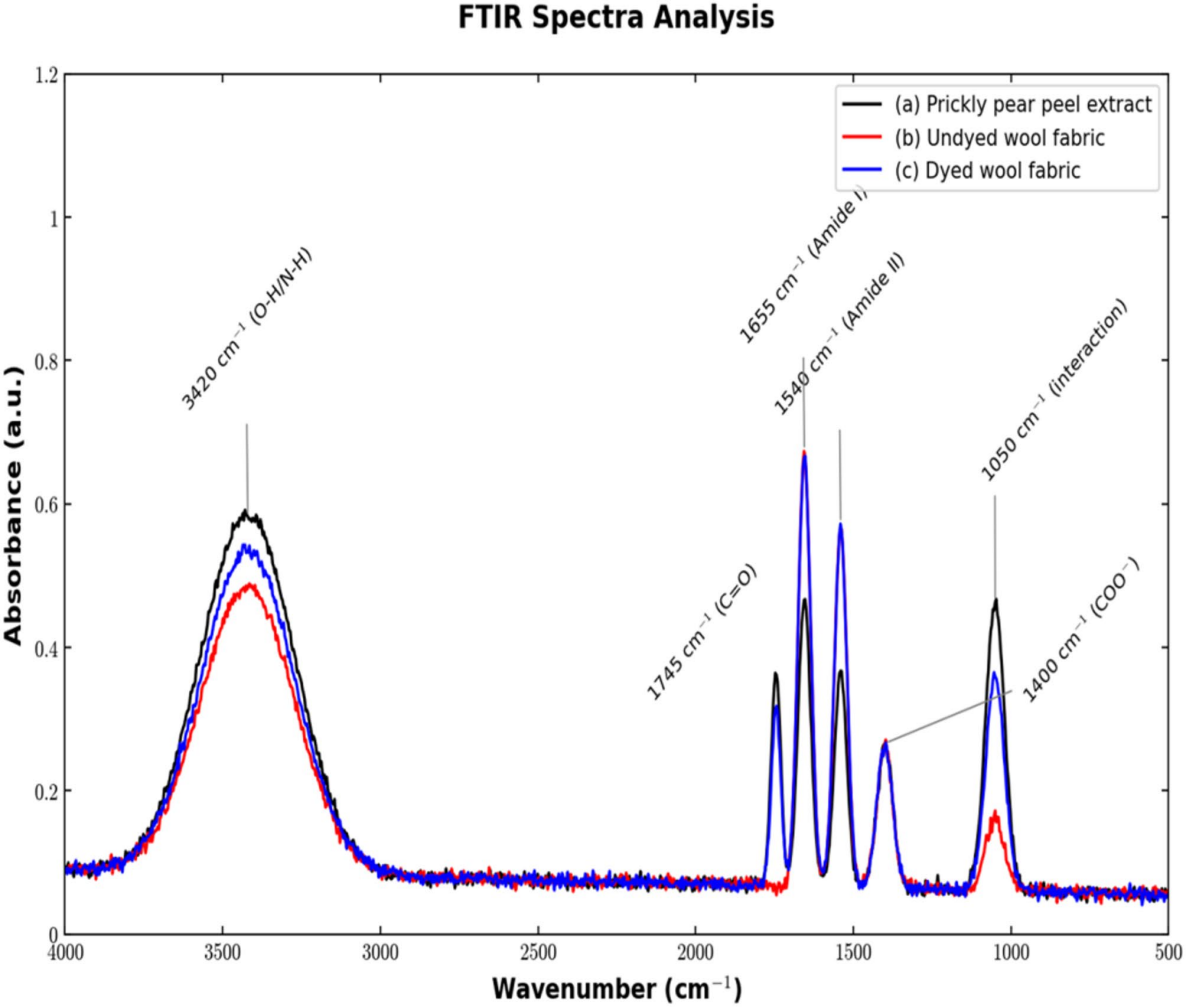
FTIR spectra of (**a**) prickly pear peel extract, (**b**) undyed wool fabric, and (**c**) microwave-dyed wool fabric.

Figure 3 presents the FTIR spectra of (a) the prickly pear peel extract, (b) undyed wool fabric, and (c) wool fabric dyed with prickly pear peel extract using the microwave-assisted method. The spectra clearly demonstrate successful attachment of betalain pigments onto the wool keratin structure. The prickly pear peel extract shows characteristic bands at 3420 cm⁻¹ (O–H/N–H stretching), 1745 cm⁻¹ (C = O stretching of carboxylic groups), and 1400 cm⁻¹ (COO⁻ symmetric stretching). In the dyed wool spectrum, these peaks appear intensified, while the amide I (1655 cm⁻¹) and amide II (1540 cm⁻¹) bands are broadened and slightly shifted, indicating hydrogen bonding and ionic interactions between the betalain carboxylate/phenolic groups and wool’s amino and amide groups. The new shoulder at 1050 cm⁻¹ further confirms dye–fiber interaction. These changes are more pronounced in the microwave-dyed sample, supporting deeper penetration and stronger fixation achieved by microwave irradiation^2^^[,3^. Generally speaking, the appearance of new peaks at 1745 cm⁻¹ (C = O of betalains) and 1400 cm⁻¹ (COO⁻), along with broadening and shifting of the amide I (1655 cm⁻¹) and amide II (1540 cm⁻¹) bands, confirms strong ionic and hydrogen-bonding interactions between betalain pigments and wool keratin. An additional interaction peak is observed at 1050 cm⁻¹.

### Effect of dyeing time

**Fig. 4:**
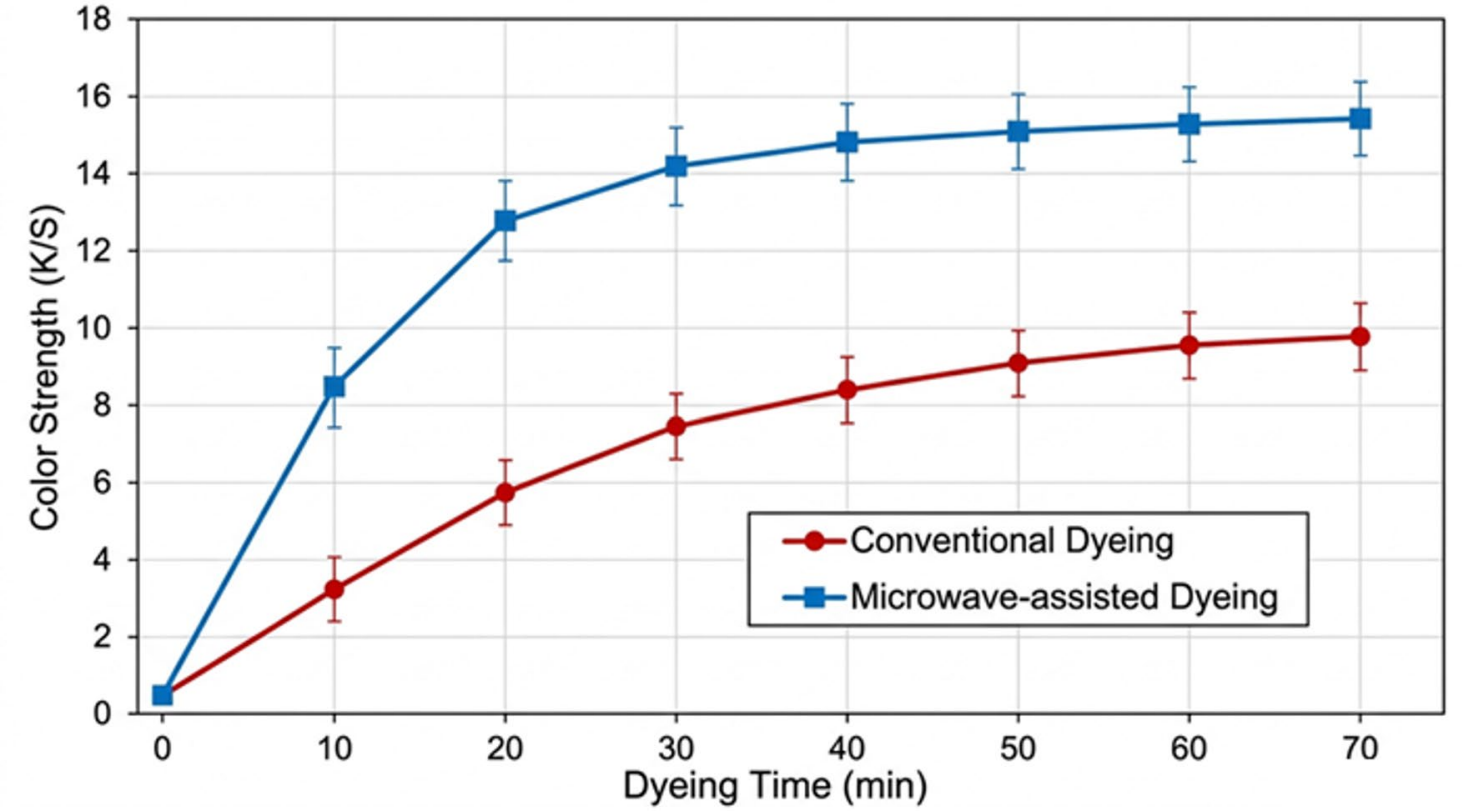
Effect of dyeing time on color strength (K/S) of wool fabrics dyed with prickly pear peel extract using conventional and microwave-assisted method (*n* = 3, mean ± SD).

Figure 4 depicts the effect of dying time on the color strength (K/S) of wool fabrics dyed with prickly pear peel extract using conventional and microwave-assisted techniques. The findings show that color strength increases with longer dyeing times in both ways, eventually reaching a plateau, which is typical of exhaustion dyeing processes in which dye uptake gradually saturates the available fiber sites^36^^23^. Notably, the microwave-assisted approach generates significantly higher K/S values in much less time than the conventional method. For example, microwave irradiation achieves optimal color strength in 3–20 min, whereas the conventional procedure takes 15–60 min or longer to get comparable depths. This improvement can be due to microwave energy’s rapid and uniform volumetric heating, which speeds dye penetration into wool fibers, enhances fiber swelling, and boosts molecular mobility of the natural colorants (betalains) found in prickly pear extract^24^^38^ Microwave irradiation causes dipole rotation and ionic conduction, resulting in faster dye breakdown and increased penetration without excessive thermal degradation^17^. In contrast, traditional heating relies on slower conductive heat transmission, which leads to gradual dye fatigue and reduced overall efficiency. These findings highlight the potential of microwave-assisted dying as an energy-efficient and time-saving option for natural dye application on wool, in line with sustainable textile processing aims^39^.

The rapid kinetics observed in microwave assisted dyeing are due to the direct interaction of microwaves with polar molecules in the dye bath and fiber. This volumetric heating mechanism provides a more efficient energy transfer compared to the surface-to-core heat transfer in conventional methods. As a result, the activation energy barrier for dye diffusion into the wool fiber is overcome more quickly, leading to faster dye uptake and equilibrium^40^. Studies by Barani et al. and Adeel et al. have consistently shown that microwave treatment significantly reduces the time required to achieve maximum dye exhaustion and fixation for natural dyes on wool, often by 60–70% compared to conventional heating^2^^18^. This accelerated process minimizes the overall dyeing cycle, leading to energy savings and increased productivity.

While not explicitly detailed in a separate figure, the temperature of the dye bath is an inherently critical parameter influencing the dyeing process, particularly in conjunction with dyeing time. The microwave-assisted process inherently involves rapid temperature elevation to optimal levels. The dyeing temperature plays a crucial role in determining the rate of dye diffusion and the swelling of wool fibers. For natural dyes on wool, an optimum temperature range (typically 70–90 °C) is essential to achieve the glass transition temperature (Tg) of the wool fiber. At this temperature, the amorphous regions of the wool become more accessible, allowing for greater penetration of dye molecules^20^. Microwave heating rapidly brings the dye bath and fiber to this optimal temperature, ensuring uniform heating throughout the material. However, exceeding the optimal temperature can lead to thermal degradation of heat-sensitive natural pigments (like betalains) and potential hydrolytic damage to the wool keratin, compromising both color quality and fiber integrity^41^(5). It has been emphasized that the importance of precise temperature control in natural dyeing to balance dye uptake with fiber preservation, a balance that microwave technology facilitates through its rapid and controlled heating capabilities^35^.

### Effect of pH

**Fig. 5:**
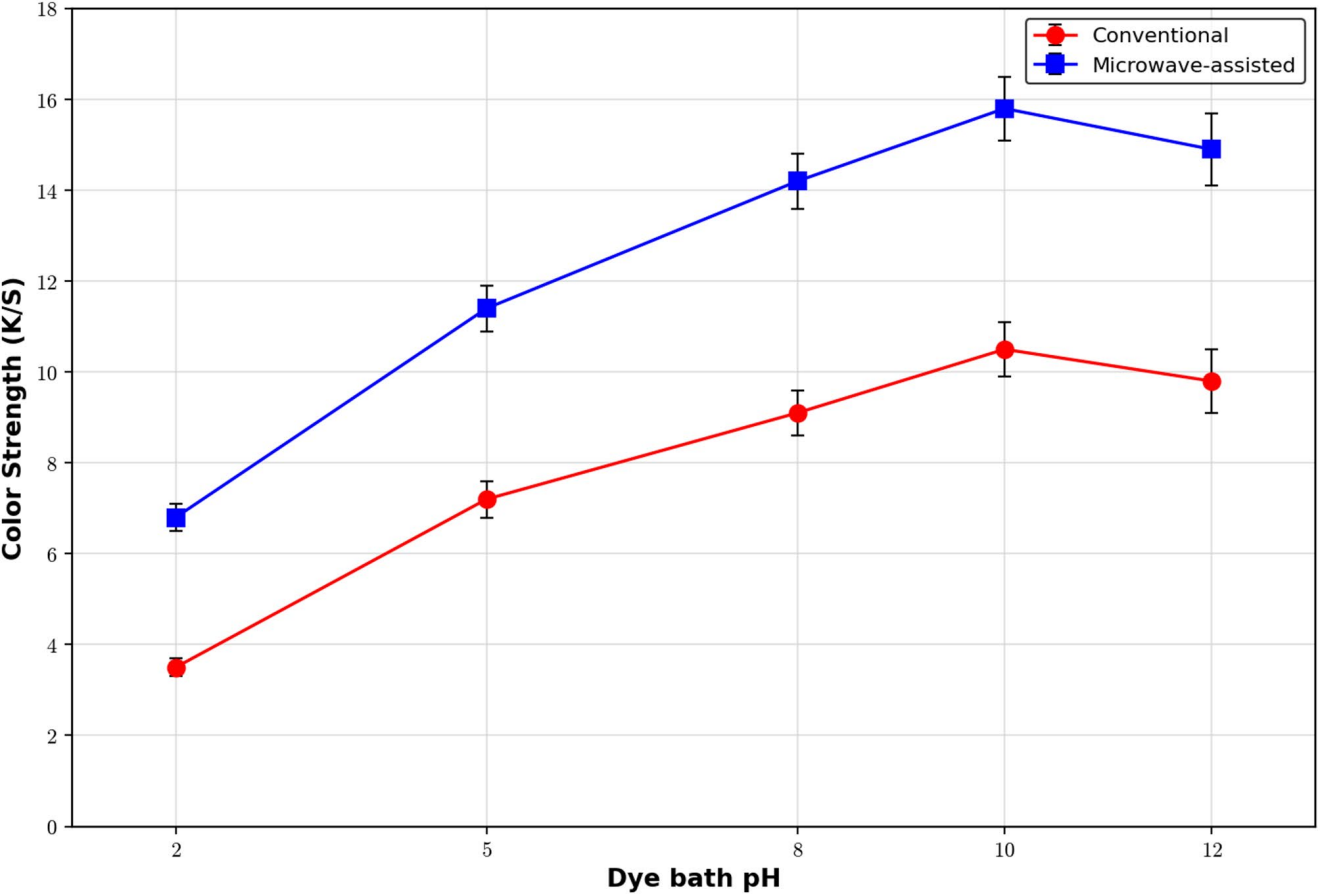
Effect of Dye Bath pH on Color Strength (K/S) of wool fabrics dyed with prickly pear peel extract using conventional and microwave-Assisted methods (*n* = 3, mean ± SD).

Figure 5 shows the change in color strength (K/S) of wool fabrics dyed with prickly pear peel extract as a function of dye bath pH using both conventional and microwave-assisted procedures. The data indicate a clear pH dependence, with K/S values increasing significantly as the medium transitions from acidic (pH ≈ 4) to alkaline conditions, with the highest color depth in the alkaline range. This trend affects not only K/S values but also color shades, with CIE L*a*b* coordinates showing darker, more saturated hues (lower L*, higher a* and b*) at optimal pH. This pattern holds true for both dyeing procedures, while microwave-assisted dyeing consistently produces higher color strength over the pH spectrum, most likely due to increased dye transport and fiber swelling caused by fast volumetric heating^23^^42^. The observed increase in color strength with increased pH can be due to the bathochromic and hyperchromic effects typical of betalain pigments (primarily betaxanthins responsible for the yellow-orange hues in prickly pear peel extracts). Betalains’ structural rearrangements are pH-sensitive: in acidic conditions, protonation dominates, resulting in lighter yellow shades, whereas deprotonation and potential condensation reactions in neutral to alkaline media enhance conjugation in the chromophore, resulting in deeper and more intense coloration^28^^43^. This result is consistent with the halochromic behavior of betalains, in which alkaline conditions promote the development of more colorful species or stabilize quinonoid structures^18^. Furthermore, wool’s isoelectric point (about pH 4–5) indicates that at higher pH, the fiber bears a negative charge, potentially lowering electrostatic repulsion with anionic dye components and allowing for better uptake^44^. These studies demonstrate the adaptability of prickly pear peel extract as a natural dye, allowing for tunable hues ranging from pale yellow in acidic baths to deeper golden tones in alkaline ones, while microwave aid increases efficiency without losing pH responsiveness.

The pH of the dye bath is a critical factor that influences the ionization state of both the wool fiber and the betalain dye molecules, thereby dictating their interaction and uptake. Wool, being an amphoteric protein fiber, has an isoelectric point (IEP) around pH 4.5. Below its IEP, wool carries a net positive charge, favoring interaction with anionic dyes. Above its IEP, it carries a net negative charge. For betalain pigments, their stability and color are highly pH-dependent. In acidic conditions, betalains are protonated, often leading to lighter shades or even degradation. As the pH increases towards neutral or alkaline conditions, deprotonation occurs, which can enhance the conjugation system of the chromophore, leading to deeper and more intense coloration (bathochromic and hyperchromic effects)^45^. While a higher pH might increase the negativecharge on wool, potentially leading to electrostatic repulsion with anionic dye components, the strong hydrogen bonding and ionic interactions formed between the betalain’s carboxylate/phenolic groups and the wool’s functional groups, especially under microwave conditions, can overcome this repulsion^46^. Ghanmi et al. (2022) highlighted that precise pH control is essential for optimizing both color yield and the stability of natural dye chromophores on wool^47^. The microwave-assisted process, by promoting rapid and uniform interaction, ensures that the optimal pH conditions are effectively utilized for superior dye uptake and color development.

**Fig. 6:**
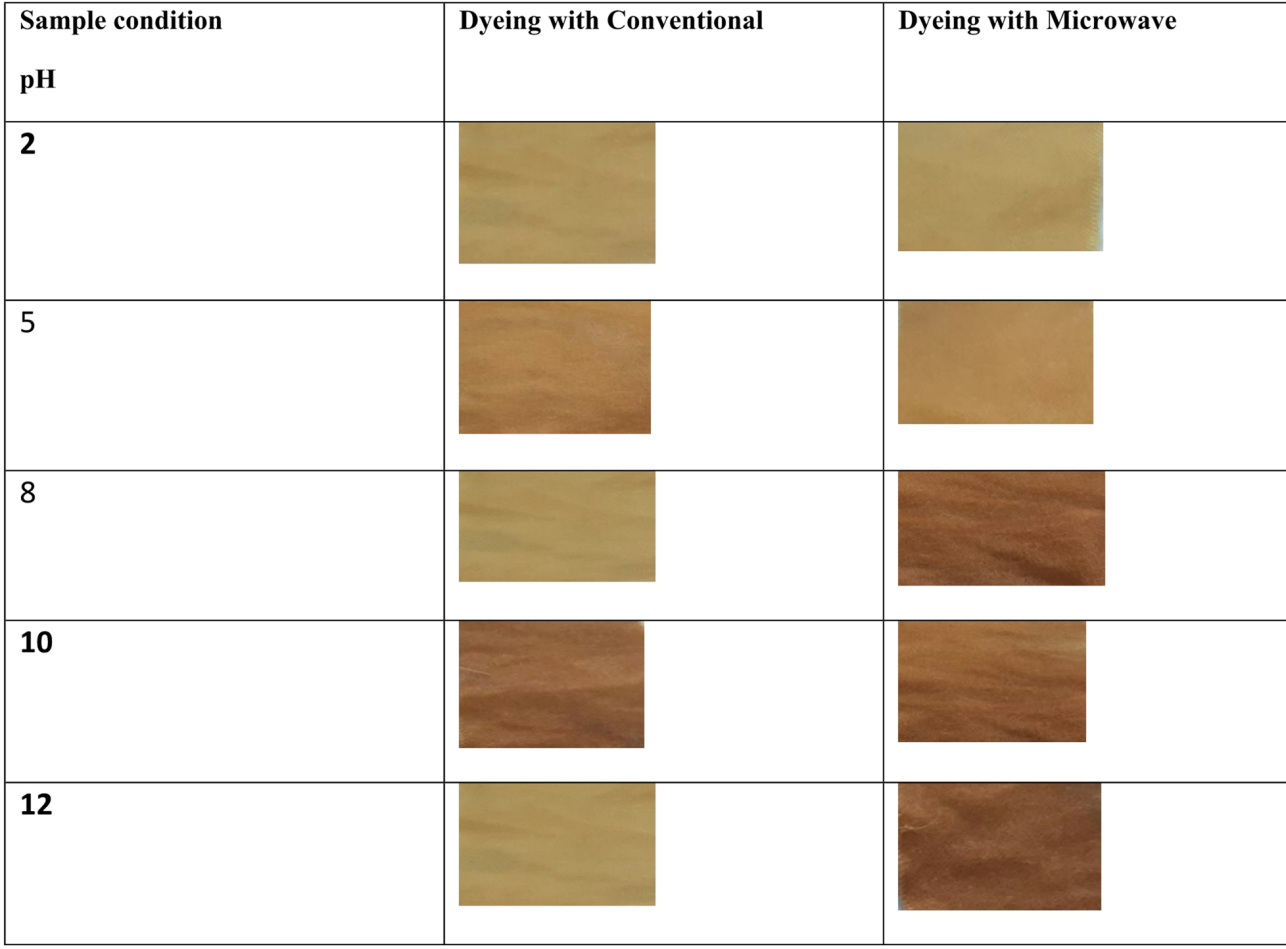
Visual appearance of wool fabrics dyed with prickly pear peel natural dye under various pH.

Figure 6 depicts images of wool fabrics colored with prickly pear peel extract at various pH levels (2, 5, 8, 10, and 12) using both conventional and microwave-assisted processes. The photos clearly show that microwave-dyed samples have deeper, more intense, and consistent coloration than conventionally dyed samples at all pH levels. This increased optical depth in microwave-treated materials is consistent with the stronger dye uptake and faster diffusion made possible by microwave energy^23^^42^^44^. Furthermore, the images demonstrate the actual impact of pH adjustment on shade variation, going from light yellow in very acidic conditions to rich golden-yellow tones in alkaline medium, proving the extract’s effective color-tuning capabilities under both dyeing processes^48^.

Conditions by Conventional and Microwave Techniques Microwave irradiation produces visually superior dyeing due to homogeneous volumetric heating, which improves pigment penetration into wool cuticles while avoiding surface aggregation found with conventional heating. Conventional samples exhibit unevenness due to slower heat transmission, particularly at pH extremes of 2 and 12. These data are consistent with increased exhaustion and fixing in microwave procedures, decreasing unfixed dye.

### Colorimetric data (L*, a*, b*)

**Fig. 7:**
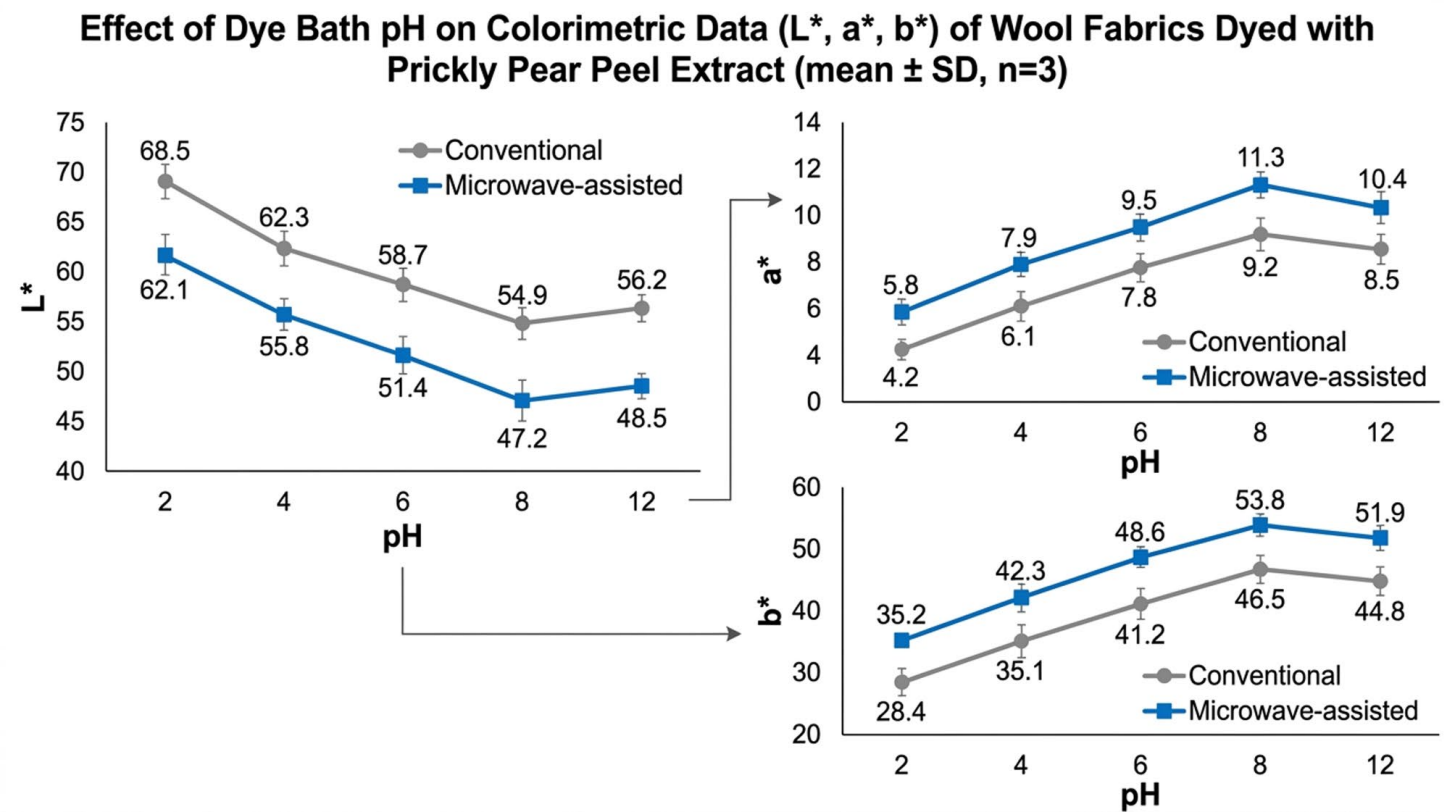
Effect of dye bath pH on the colorimetric data values of wool fabrics dyed with prickly pear peel extract using conventional and microwave methods (*n* = 3, mean ± SD).

Figure 7 depicts how dye bath pH influences the CIE L*, a*, and b* values of wool fabrics dyed with prickly pear peel extract using both traditional and microwave-assisted methods. As the pH rises from acidic to alkaline, both dyeing methods exhibit a consistent fall in lightness (L*), implying deeper hues. The b* values (yellow-blue axis) increase significantly with rising pH, indicating a shift toward more intense yellow hues. The a* values (red-green axis) remain positive but exhibit only mild increases, indicating a minor contribution from reddish tones in alkaline media. Across the pH range, microwave-dyed samples have lower L* (darker hues) and higher b* values than conventionally colored samples, indicating that microwave irradiation improves dye uptake and color intensity^23^^49^. These colorimetric alterations correspond to the pH-sensitive chromophoric system of betalains, notably betaxanthins, present in prickly pear peel extract. Higher pH promotes deprotonation, which enhances electron delocalization, resulting in less brightness and more yellowness^44^^50^. The statistics confirm the eye observations and color strength measurements, demonstrating that alkaline dyeing conditions provide richer, more vivid golden-yellow hues on wool.

### Dyeing performance: Exhaustion (E %) and fixation (F%)

**Fig. 8:**
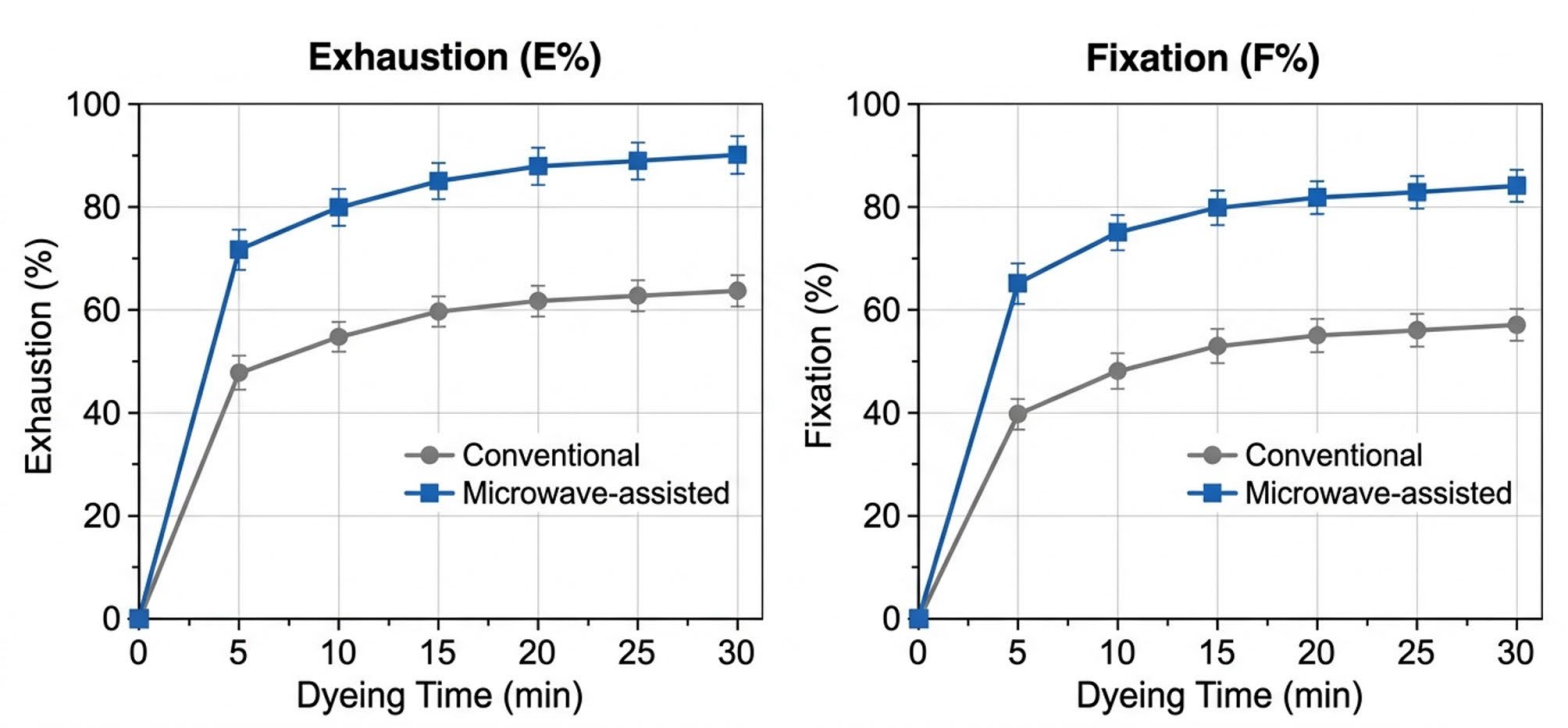
Exhaustion (E%) and fixation (F%) of wool fabrics dyed via conventional and microwave-assisted processes with prickly pear peel extract at different dyeing times (*n* = 3, mean ± SD). Microwave values are significantly higher (*p* < 0.01, Student’s t-test).

Figure 8 shows a considerable improvement in both exhaustion (E%) and fixation (F%) when the microwave-assisted procedure is used instead of the standard approach for dyeing. Natural dyes, such as those derived from Prickly Pear Peel (Opuntia ficus-indica), frequently encounter low affinity and slow diffusion rates in standard aqueous media^22^. The statistics show that microwave irradiation significantly shortens the time required to reach high tiredness levels. For example, within 30 min, the microwave-assisted technique achieves fatigue and fixing rates substantially exceeding those of the conventional process over the same time span. This enhancement is due to the volumetric heating and ionic conduction effects of microwave radiation. Unlike traditional heating, which relies on slow thermal conduction from the surface to the core, microwaves interact immediately with the polar molecules (water and color) and the wool fiber itself^4^. This rapid energy transfer increases the kinetic energy of the dye molecules, allowing them to diffuse more quickly into the amorphous portions of the wool fiber^38^. Furthermore, microwave radiation can promote “micro-boiling” inside the fiber structure, thereby expanding the pores and increasing the available surface area for dye-fiber interaction^24^.

**Fig. 9:**
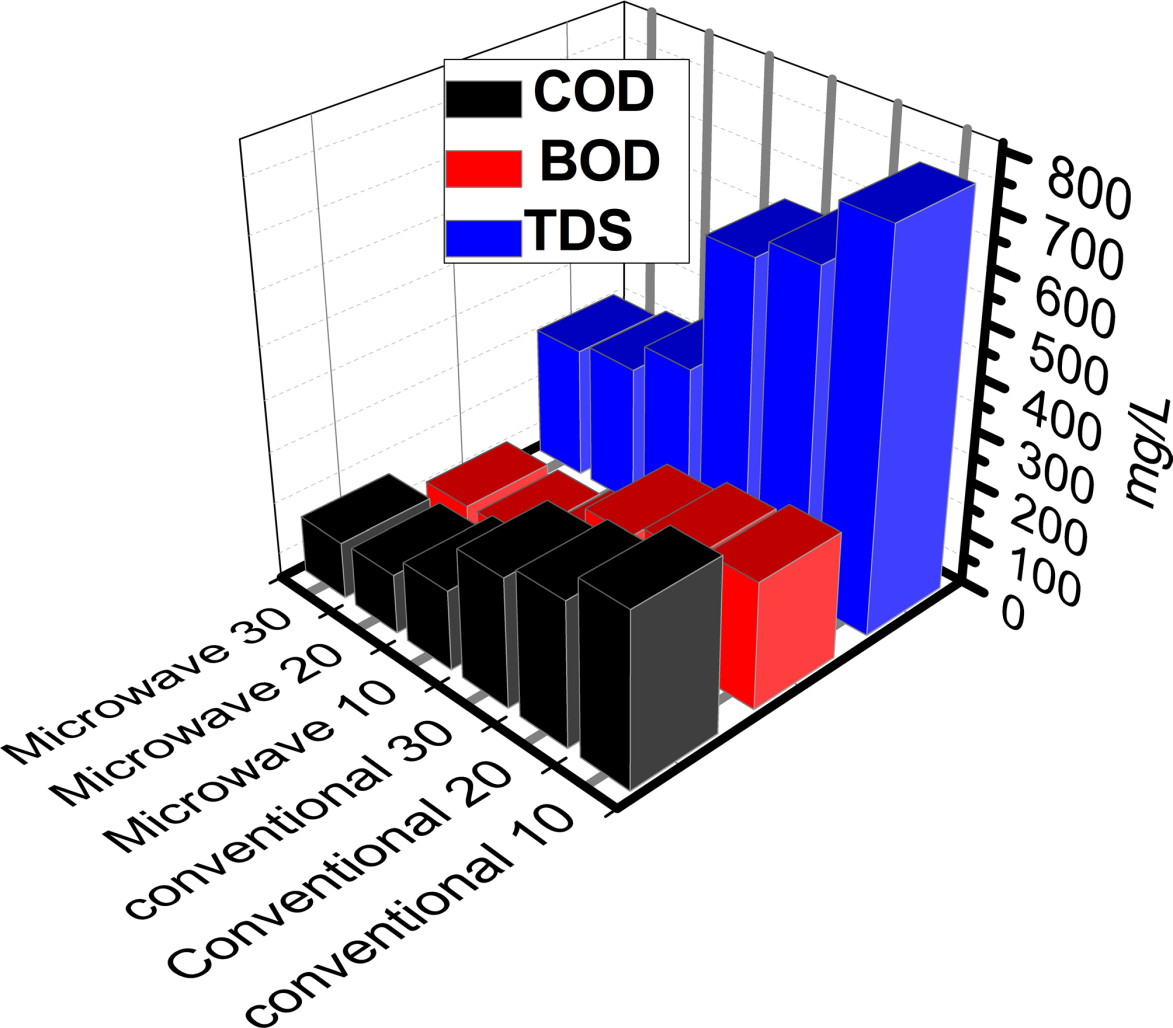
Comparison of BOD, COD, and TDS of discharged water between conventional and microwave-assiste dyeing processes with prickly pear peel extract.

### Mordant–dye–fiber interactions in conventional dyeing

**Scheme 1:**
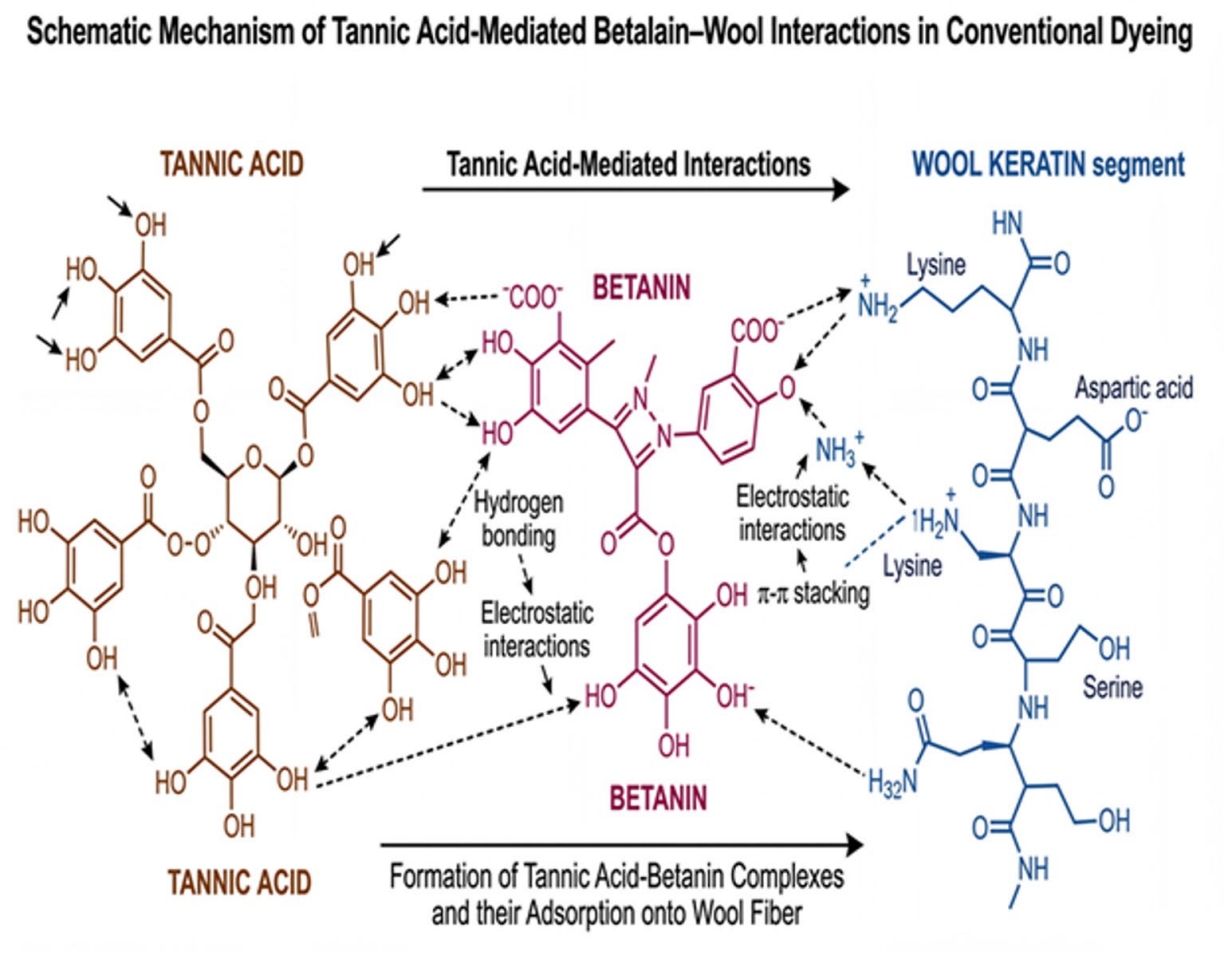
Schematic mechanism of tannic acid-mediated betalain–wool interactions in conventional dyeing.

Tannic acid functions as a bridging mordant, creating numerous hydrogen bonds, electrostatic (ionic) interactions, and coordination complexes between its phenolic hydroxyl groups, betanin’s carboxylate (–COO⁻) and phenolic –OH groups, and wool keratin’s amino (–NH₂/–NH₃⁺), carboxyl (–COO⁻), hydroxyl (–OH), and amide (–CO–NH–) groups. Tannic acid serves as a multipurpose bridge that improves dye fixation in the traditional approach, as shown in Scheme 1. Its many phenolic –OH groups form strong hydrogen bonds with the carboxylate and phenolic groups of betanin, as well as with the amide carbonyls and side-chain –OH/–NH₂ groups of wool keratin.

The negatively charged carboxylate groups of betanin and the protonated lysine residues (–NH₃⁺) of wool also interact electrostatically (ionic), and the dye–mordant–fiber assembly is further stabilized by coordination complexes and π-π stacking. A strong interfacial network is produced by these multi-site interactions, which enhance fastness and color yield^51^^[,52^. The microwave-assisted method, which is carried out without the use of a mordant, shows noticeably greater color strength and fixation even if tannic acid offers efficient bridging in the traditional method. There are multiple reasons for this exceptional performance: (1) rapid volumetric heating that accelerates dye diffusion and allows deeper penetration of betalain molecules into the swollen wool fiber interior; (2) enhanced fiber swelling under microwave irradiation that exposes a greater number of accessible binding sites on keratin; (3) direct ionic and hydrogen-bonding interactions between betanin carboxylate/phenolic groups and wool functional groups without the need for a mordant intermediary; and (4) shorter processing time combined with uniform energy distribution, which minimizes dye aggregation and thermal degradation of the betalain chromophore. Consequently, the microwave route achieves more efficient and stable dye–fiber attachment compared to the conventional mordanted method^51^^[,53^^[,54^.

### Fastness properties

Table 3 highlights the fastness qualities (washing, perspiration, rubbing, and light) of wool garments dyed with prickly pear peel extract at various pH levels using traditional and microwave-assisted processes. Overall, the dyed samples have good to very good fastness ratings under all testing conditions, with scores ranging from 4 to 4–5 (on the ISO grey scale) for washing, perspiration, and rubbing, and 6 to 7 (on the blue wool scale) for light fastness.

**Table 3:** The UPF of wool fabrics dyed with prickly pear peel extract using conventional and microwave methods (n = 3, mean ± SD)

No1	AS/NZ S4399:1996-UPF	AS/NZ S4399:1996-UVA Transmittance	AS/NZ S4399:1996-UVB Transmittance	AATCC Test Method 183:2010-UPF	AATCC Test Method 183:2010-UVA Transmittance	AATCC Test Method 183:2010-UVB Transmittance
Blank	31.0 ± 1.2	1.2	7	31.5 ± 1.1	1.1	7
Dyed Microwave	59.5 ± 2.1**	2.1	2.8	60.0 ± 1.8**	2.1	2.8
Dyed Conventional	39.2 ± 1.5*	1.8	2.9	39.5 ± 1.4*	1.8	2.8

(p < 0.05, *p < 0.01 vs. undyed, t-test)

Microwave-dyed samples consistently perform slightly better or equal to conventionally dyed samples, especially in alkaline pH conditions (pH 8–12), where ratings for washing (Alt and StW/StS), perspiration (acidic and basic), and rubbing (dry and wet) are frequently 4–5. This enhancement can be due to faster penetration and greater physical/chemical interactions between betalain pigments and wool keratin during rapid microwave heating, resulting in improved fixing within the fiber matrix^23^^[,49^. Enhanced dye-fiber bonding reduces dye migration during future wet treatments. Light fastness is satisfactory^55^^[,56^ for both processes and over the pH range, with some alkaline-dyed microwave samples showing somewhat higher values (up to 7). This moderate to good light fastness corresponds to the known behavior of natural betalains, which, while less photostable than some synthetic dyes, benefit from the protective environment given by wool’s protein structure and the absence of significant photosensitizing chemicals^60^.

The fastness results show that, when applied under optimal conditions, prickly pear peel extract can make environmentally friendly colored wool with commercially acceptable durability, particularly when microwave aid is used. These results are consistent with prior research on natural cactus pear dyes, which found good to exceptional fastness qualities on wool when mordants were applied or under controlled pH settings^18^^[,55^.

### Effluent comparison

Sustainable textile production requires consideration of the dyeing process’s environmental impact. Figure 8 shows a clear comparison of three important wastewater parameters: biological oxygen demand (BOD), chemical oxygen demand (COD), and total dissolved solids. The results show that the microwave-assisted technique greatly decreases pollutants in released wastewater. Chemical Oxygen Demand (COD) and Biological Oxygen Demand (BOD) are the main indices of organic contamination in textile wastewater. High COD/BOD levels in conventional dyeing are frequently caused by unexhausted dye molecules and auxiliary chemicals lingering in the dye solution61. The lower COD and BOD values observed in the microwave assisted process are directly correlated firstly, with the higher exhaustion rates shown in Fig. 7. Because a greater proportion of the betalain pigments from the prickly pear extract is fixed onto the wool fabric, fewer organic contaminants are discharged into the effluent56 ; secondly, and critically, the complete omission of tannic acid mordant in the microwave process, whereas the conventional dyeing employed 4% tannic acid (owf) via simultaneous mordanting. Tannic acid, a polyphenolic compound, contributes additional organic matter that increases oxygen demand and dissolved solids when released into the wastewater. TDS represents the concentration of dissolved inorganic and organic substances in the water. In traditional dyeing, substantial doses of salts or leveling agents are frequently necessary to achieve fatigue, which significantly raises TDS levels62. The microwave-assisted approach lowers the need for such chemical additions by increasing the dye’s inherent affinity for the fiber through physical activation, resulting in a cleaner discharge with reduced TDS62. The use of microwave technology in natural dyeing of wool with Prickly Pear Peel Extract constitutes a significant step toward sustainable colors. The procedure not only optimizes technical parameters such as exhaustion and fixation, but it also meets global environmental standards by significantly lowering organic and inorganic loads in wastewater. This combination of efficiency and ecological makes microwave-assisted dyeing a viable alternative to traditional energy-intensive and polluting processes.

### Ultraviolet protection factor (UPF)

Table 2 shows the ultraviolet protection factor (UPF) and UV transmittance (UVA and UVB) values for undyed (blank) and dyed wool fabrics, as determined by both AS/NZS 4399:1996 and AATCC Test Method 183:2010 standards. The undyed wool fabric has a baseline UPF of roughly 31-31.5, indicating that it provides “very good” UV protection (UPF 25–39) due to the natural UV-absorbing characteristics of wool keratin and its comparatively dense structure. After dyeing with prickly pear peel extract, UV protection improves significantly. Microwave-dyed samples had a high UPF of 59.5–60, equivalent to an “excellent” protection category (UPF 50+), whereas conventionally colored samples show a small increase to 39.2–39.5 (still “very good”). The increased UPF in colored fabrics is mostly due to the presence of betalain pigments (betaxanthins and minor betacyanins) from prickly pear peel extract, which have strong UV-absorbing properties, particularly in the UVA and UVB regions^18^^[,63^. These pigments work as natural UV filters, absorbing and dispersing UV rays to reduce transmittance through the cloth. The much higher UPF in microwave-dyed samples is associated with increased dye uptake and deeper color strength, resulting in more effective pigment deposition within the fiber matrix^22^^[,57^^[.14^. The reduced UVA and UVB transmittance values in dyed samples (particularly microwave-dyed samples) demonstrate the better barrier characteristics against ultraviolet radiation. These results show the multifunctional usefulness of prickly pear peel extract as a natural dye, delivering not only attractive coloration but also outstanding UV-protective performance to wool garments. Microwave-assisted dying provides the most effective improvement.

**Table 4:** Antibacterial activity of undyed and microwave-dyed wool fabrics (mean ± standard deviation, n = 3).

Sample	Staphylococcus aureus (ATCC 6538)	Escherichia coli (ATCC 8739)
Undyed wool fabrics	20 ± 2.1%^*^	40 ± 3.4%
Dyed wool with Microwave	80 ± 1.8%	85 ± 2.3%

* ﻿Statistical analysis (one-way ANOVA followed by Tukey’s post-hoc test) showed that the reduction percentages for the microwave-dyed samples were significantly higher than the undyed control (p < 0.01).

### Antibacterial activity

Table 4 presents the quantitative antibacterial activity of undyed and dyed wool fabrics against Staphylococcus aureus (Gram-positive) and Escherichia coli (Gram-negative), as measured using AATCC Test Method 100–1999. Undyed wool has poor antibacterial properties, with bacterial reduction rates of 20% and 40% against S. aureus and E. coli, respectively. In contrast, wool fabrics dyed with prickly pear peel extract using a microwave-assisted process show much higher reduction rates of 80% against S. aureus and 85% against E. coli. This significant increase in antibacterial efficiency is related to the bioactive chemicals found in prickly pear peel extract, particularly betalains (betaxanthins and betacyanins), which are well-documented for their antioxidant and antimicrobial effects^57^^[,61^**.** These compounds can disrupt bacterial cell membranes, impede enzyme activity, and produce reactive oxygen species that harm microbial structures. Microwave-dyed samples function better because they have stronger dye fixation and deeper penetration of bioactive pigments into the wool fibers, resulting in better interaction with bacteria^22^^[,38^. These findings show that dyeing wool with prickly pear peel extract not only adds color but also has significant antibacterial properties, making it a promising eco-friendly method for generating protective and hygienic fabrics.

Considering the earlier results on the comprehensive analysis of the dyeing processes using prickly pear peel extract on wool fabrics few points may be concluded (a) Enhanced Dyeing Efficiency: The integration of microwave technology significantly optimizes the dyeing of wool with Prickly Pear Peel Extract, achieving superior exhaustion (E %) and fixation (F %) in a fraction of the time required by conventional methods. This is attributed to the volumetric heating and ionic conduction effects of microwaves, which accelerate dye diffusion into the proteinous fiber matrix. ; (b) superior Functional Finishing: The dyed wool fabrics exhibit multifunctional properties, most notably achieving an “Excellent Protection” (UPF 50+) rating and significant antimicrobial activity against both Staphylococcus aureus and Escherichia coli. The microwave-assisted process ensures a denser and stable retention of bioactive betalains and phenolics compared to traditional thermal processing; (c) high Technical Fastness: The prickly pear colorants demonstrate remarkable stability on wool, with light fastness ratings reaching 6–7 and washing/perspiration fastness consistently rated between 4 and 4–5. This indicates that the natural pigments form robust chemical bonds with the wool’s amino acid side chains, ensuring durable coloration; (d) eco-Friendly and Sustainable Process: The microwave-assisted approach represents a green leap in textile processing by drastically reducing the pollution load in discharged effluent. Significant reductions in BOD, COD, and TDS levels were observed, aligning the process with global sustainability standards and minimizing the environmental footprint of natural dyeing; (e) optimized pH Dependency: The technical and functional performance of the prickly pear extract is highly dependent on the dye bath pH, with optimum results generally achieved in the acidic to neutral range (pH 4–6). This range facilitates the ideal ionic balance for maximum dye-fiber affinity and functional bio-active retention.

Although the results of this study are promising, several limitations should be acknowledged. The experiments were performed at laboratory scale using a domestic microwave oven, which may not fully reflect conditions in industrial microwave systems. Only one type of wool fabric (Australian merino) was investigated, and the long-term durability of both color and functional properties (UV protection and antibacterial activity) under repeated laundering, light exposure, and storage conditions was not evaluated. Furthermore, the color palette obtained was primarily in the yellow-orange range. Future studies should address process scale-up, reproducibility across different wool types and larger fabric batches, expansion of shade gamut using bio-mordants, and comprehensive life-cycle assessment to better evaluate industrial applicability and overall environmental benefits.

## Conclusions

In conclusion, this study demonstrates the successful valorization of prickly pear (*Opuntia ficus-indica*) peel waste as a natural dye source for wool fabrics. Microwave-assisted dyeing achieved higher color strength, improved dye exhaustion and fixation, and comparable or better fastness properties in significantly shorter processing time without the use of tannic acid mordant, compared to the conventional method. The dyed wool fabrics exhibited good to very good fastness properties, high ultraviolet protection (UPF up to 60), and notable antibacterial activity. Moreover, the microwave process resulted in lower effluent pollution (BOD, COD, and TDS). These results suggest that combining agricultural waste valorization with microwave technology is a promising eco-friendly strategy for producing multifunctional wool textiles. Further research on industrial scalability, long-term durability testing, and expansion of the color range is recommended to support practical implementation of this approach.

### Method

#### Materials

Australian merino wool fabric with a mean fiber diameter of 20.8 μm was kindly supplied by Misr for Spinning and Weaving Co., El-Mahalla El-Kobra, Egypt. All chemicals used were of laboratory grade.

### Extraction of the natural dye

Prickly pear (*Opuntia ficus-indica*) fruit peels were collected and washed thoroughly with running water to remove surface impurities. Five kilograms of cleaned peels were blended for 10 min to form a homogeneous thick puree. The puree was filtered through fine strainers to obtain a dark yellow filtrate. The filtrate was then diluted with distilled water to a final volume of 1 L. This stock solution served as the dyeing bath throughout the study. The extraction yield was 48.5 g of dry extract (0.97% w/w based on fresh peel weight). The total betalain concentration in the 1 L stock solution was determined spectrophotometrically at 480 nm (λ_max for betaxanthins) using the standard molar extinction coefficient of indicaxanthin (ε = 48 000 L mol⁻¹ cm⁻¹). After appropriate dilution (1:10), the absorbance was measured as 1.85. The calculated betalain concentration in the undiluted stock was **285 mg/L**. This value is consistent with literature reports for yellow prickly pear peel extracts and allows direct comparison and reproducibility with previous studies^63^.

### Dyeing procedures

#### Conventional dyeing

Dyeing was conducted using the exhaustion method. The dye bath was prepared from the extracted solution with a liquor ratio of 1:50 (fabric to liquor). The pH of the dye bath was adjusted in the range of 4.0–9.0 using acetic acid (for acidic media) or sodium hydroxide (for alkaline media). The temperature was raised to 85 °C, and the wool fabric was immersed and dyed for 15–60 min. After dyeing, the samples were rinsed with warm water (≈ 40 °C), followed by cold running water, and air-dried at room temperature. For simultaneous mordanting, tannic acid was added directly to the dye bath at a concentration of 4% (on weight of fabric, owf) under optimized conditions, following the procedure reported by Ali and El-Mohamedy^18^^[,19^, where this concentration yielded the highest color strength and uniform dyeing.

### Microwave-assisted dyeing

Microwave-assisted dyeing was performed using the exhaustion technique in a domestic microwave oven (Samsung, 700 W maximum power output, 2.45 GHz frequency). The dye bath was prepared as described in Sect.  2.3.1 with a liquor ratio of 1:50. Fabric samples (5 × 10 cm) were immersed in the dye solution inside a 250 mL Pyrex beaker placed at the center of the rotating turntable to ensure uniform exposure. Irradiation was applied in intermittent mode (30 s on / 10 s off cycles) for a total effective time of 3–20 min. This pulsed operation prevented overheating and allowed precise control of the internal temperature, which was continuously monitored using a non-contact digital infrared thermometer (accuracy ± 1 °C) and maintained at 85 ± 2 °C by adjusting the on/off cycles. After dyeing, the fabrics were removed, rinsed with warm water (≈ 40 °C), and air-dried at room temperature. No mordants were used in this process to enable direct comparison with the conventional mordant-assisted method.

### Measurements and evaluations

Color strength (K/S) was measured at the wavelength of maximum absorbance using an SF600+-CT Datacolor spectrophotometer. The color coordinates of the dyed fabrics were determined under the CIE L*a*b* system (International Commission on Illumination) with a HunterLab ColorQuest II spectrophotometer, adhering to CIE 1931 specifications. Measurements were conducted with a D65 light source, a 10° viewing angle, specular reflection incorporated, a 30 mm aperture, and in reflectance configuration. Data processing for color evaluation was performed using Premier Colorscan software^20^. Fastness properties were evaluated according to ISO standards: ISO 105-C06 (2010) for washing, ISO 105-E04 (2013) for perspiration (acidic and alkaline), ISO 105-B02 (2014) for light, and ISO 105-X12 for rubbing. The ultraviolet protection factor (UPF) was determined according to AATCC 183–2004 and ASTM D6603-00. Fabric thickness was measured per ASTM D1777, and weight per unit area per ASTM D3776. Molecular analysis of wool, dye and dyed wool fabrics were analyzed by Fourier-transform infrared spectroscopy (FTIR, PerkinElmer Spectrum BX) in KBr tablets (1:9 material: KBr mix). Antibacterial activity was assessed quantitatively against *Staphylococcus aureus* (ATCC 6538, Gram-positive) and *Escherichia coli* (ATCC 8739, Gram-negative) using AATCC Test Method 100–1999. Bacterial cultures were prepared to an inoculum density of approximately **1.0 × 10⁵ CFU/mL**. Circular fabric samples (4.8 cm diameter, ≈ 0.5 g) were placed in sterile jars containing 50 mL of the bacterial suspension and incubated at 37 °C for **24 h** contact time. After incubation, the surviving bacteria were eluted, serially diluted, and plated on nutrient agar. All experiments were performed in **triplicate**. The percentage reduction in bacterial count was calculated using the formula:

$$\% {\mathrm{Reduction}} = {\text{ }}\left( {{\mathrm{A}} - {\mathrm{B}}} \right)/{\text{A }} \times {\mathrm{1}}00\backslash \%$$

Where A is the number of bacteria recovered from the undyed control fabric and *B* is the number recovered from the dyed fabric after 24 h.

### Dye exhaustion and fixation

UV-Vis spectrophotometry was employed to calculate the dye uptake percentage (%E), the degree of dye attachment (%F), and the overall attachment efficiency (%T) on the wool textiles, based on the equations below^20^^[,21^:

$$\% {\text{E }} = {\text{ 1}}00{\text{ }} \times {\text{ }}\left( {{\mathrm{A}}_{0} {\text{ }} - {\text{ A}}{}} \right){\text{ }}/{\text{ A}}_{0}$$

$$\% {\text{F }} = {\text{ 1}}00{\text{ }} \times {\text{ }}\left( {{\mathrm{A}}_{0} {\text{ }} - {\text{ A}}{}{\text{ }} - {\text{ A}}{}} \right){\text{ }}/{\text{ }}\left( {{\mathrm{A}}_{0} {\text{ }} - {\text{ A}}{}} \right)$$

$$\% {\text{T }} = {\text{ }}\left( {\% {\text{E }} \times {\text{ }}\% {\mathrm{F}}} \right){\text{ }}/{\text{ 1}}00$$

In these formulas, A₀ represents the initial absorbance of the dyeing solution, A₁ is the absorbance following the dyeing step, and A₂ is the absorbance after the washing process.

### Effluent treatment

To evaluate and contrast the environmental impact of the traditional dyeing and microwave-enhanced dyeing methods on wool fabrics, analyses were performed on the wastewater generated post-dyeing, focusing on chemical oxygen demand (COD), biological oxygen demand (BOD), and total dissolved solids (TDS).The COD assessment, which indicates the level of oxidizable organic matter in the wastewater, was conducted via the potassium dichromate oxidation technique using a KIT COD/1500 setup along with an APP Nanocor 300D and Nanocolor R-8 thermoreactor. BOD, reflecting the oxygen required for microbial decomposition of organic substances, was quantified with an OxiTop respirometric system (model IS 12, manufactured in Germany). TDS, expressed in milligrams per liter (mg/L), was determined through routine laboratory protocols for water quality testing. These evaluations adhered to the protocols specified in the Standard Methods^22,24^.

## Data Availability

The datasets generated and/or analyzed during the current study are available from the corresponding author upon reasonable request.
